# HPV-16 E7 expression up-regulates phospholipase D activity and promotes rapamycin resistance in a pRB-dependent manner

**DOI:** 10.1186/s12885-018-4392-8

**Published:** 2018-04-27

**Authors:** Tatiana Rabachini, Enrique Boccardo, Rubiana Andrade, Katia Regina Perez, Suely Nonogaki, Iolanda Midea Cuccovia, Luisa Lina Villa

**Affiliations:** 1 Ludwig Institute for Cancer Research - Hospital Sírio-Libanês, São Paulo, SP Brazil; 2 Departamento de Bioquímica, Instituto de Química, Universidade de São Paulo, São Paulo, SP Brazil; 3 Departamento de Microbiologia, Instituto de Ciências Biomédicas, Universidade de São Paulo, São Paulo, SP Brazil; 4 Departamento de Biofísica, Escola Paulista de Medicina, Universidade Federal de São Paulo, São Paulo, SP Brazil; 5 Centro de Patologia do Instituto Adolfo Lutz, São Paulo, SP Brazil; 6 Faculdade de Medicina da Universidade de São Paulo, Instituto do Câncer do Estado de São Paulo, São Paulo, SP Brazil

**Keywords:** HPV, E7, PLD, Phospholipase, Rapamycin, Phosphatidic acid, PA, mTOR, pRb

## Abstract

**Background:**

Human Papillomavirus (HPV) infection is the main risk factor for the development and progression of cervical cancer. HPV-16 E6 and E7 expression is essential for induction and maintenance of the transformed phenotype. These oncoproteins interfere with the function of several intracellular proteins, including those controlling the PI3K/AKT/mTOR pathway in which Phospolipase D (PLD) and Phosphatidic acid (PA) play a critical role.

**Methods:**

PLD activity was measured in primary human keratinocytes transduced with retroviruses expressing HPV-16 E6, E7 or E7 mutants. The cytostatic effect of rapamycin, a well-known mTOR inhibitor with potential clinical applications, was evaluated in monolayer and organotypic cultures.

**Results:**

HPV-16 E7 expression in primary human keratinocytes leads to an increase in PLD expression and activity. Moreover, this activation is dependent on the ability of HPV-16 E7 to induce retinoblastoma protein (pRb) degradation. We also show that cells expressing HPV-16 E7 or silenced for pRb acquire resistance to the antiproliferative effect of rapamycin.

**Conclusion:**

This is the first indication that HPV oncoproteins can affect PLD activity. Since PA can interfere with the ability of rapamycin to bind mTOR, the use of combined strategies to target mTOR and PLD activity might be considered to treat HPV-related malignancies.

**Electronic supplementary material:**

The online version of this article (10.1186/s12885-018-4392-8) contains supplementary material, which is available to authorized users.

## Background

Human papillomavirus (HPV) is the most prevalent sexually transmitted infection and a necessary cause of cervical cancer, the third most common cancer in women worldwide [1]. More than 99% of cervical cancers contain DNA of HPV high-risk types [2]. In addition, HPV DNA is also found in a significant percentage of other anogenital lesions as well as oral and oropharyngeal tumors [3–5]. HPV-16 is the most prevalent type and it is found in almost 50% of all cervical cancer cases [6]. The two major HPV oncoproteins, E6 and E7, are consistently expressed in all HPV positive cancers. The expression of these proteins in primary human keratinocytes effectively induces their immortalization [7]. Furthermore, when grown under conditions that allow stratification and the formation of skin-like structures, cells immortalized with E6 and E7 from high-risk HPV types display morphological hallmarks of high-grade squamous intra-epithelial lesions, well-established precursors of cervical cancer [8]. The best characterized activity of HPV-16 E6 and E7 is their ability to bind to and induce the proteasome-mediated degradation of tumor suppressors p53 and pRb, respectively [9, 10]. Besides, both HPV-16 E6 and E7 are able to bind to and alter the biological function of several other cellular proteins [11]. Among them are many members of the phosphatidylinositol (PI)-3-kinase (PI3K)/Akt/mammalian target of rapamycin (mTOR) signaling pathway [12]. The PI3K/Akt/mTOR signaling axis plays a very important role in HPV-induced carcinogenesis by acting through multiple cellular and molecular events [13]. Although the regulation of mTOR through PI3K/AKT has been extensively described, another mechanism contributing to activation of mTOR has been proposed. Phosphatidic acid (PA), a product of phospholipase D, is required for mTORC1 activation by mitogens as well as amino acid signals [14–16]. More recently, PA was identified as a major product capable of displacing DEPTOR, a mTOR binding protein that normally functions to inhibit both mTORC1 and mTORC2 pathways [17]. PA species with unsaturated fatty acids chains, such as those produced by PLD, bind with high affinity to the FRB domain of mTOR in a manner that is competitive with its inhibitor rapamycin. As a consequence, elevated PLD activity has been associated to rapamycin resistance [18, 19]. In addition, phospholipase D enzymes play a fundamental role in cells: they maintain the integrity of cellular membranes and they participate in cell signaling including cytoskeletal dynamics, cell migration, intracellular protein trafficking, and cell proliferation. Consistent with this data, increased PLD activity has been reported in a large number of human cancers, including breast, colon, gastric, and kidney [20].

Our results show that upon HPV-16 E7 expression, primary human foreskin keratinocytes upregulate PLD protein levels and activity. Such effect is dependent on the integrity of the E7 LxCxE binding motif and, ultimately to the ability of HPV-16 E7 to induce pRb degradation and promote immortalization. We also show that organotypic cultures of keratinocytes expressing HPV-16 E7 become resistant to the antiproliferative effect of rapamycin.

## Methods

### Cell culture

Low passage-pooled neonatal foreskin keratinocytes or Primary Human Keratinocytes (cat no. 192906) (Lonza Walkersville, Inc., Walkersville, MD) were maintained in K-SFM media supplemented with 5 μg/L EGF and 50 mg/L bovine pituitary extract (BPE) (Invitrogen, Carlsbad, CA, USA).

### Recombinant retroviruses and retroviral-mediated gene transfer

Retroviral vectors pLXSN-neo, pLXSN-E6, −E7, −E6E7, E7E26G and -E7CVQ68-70AAA were kindly provided by Dr. Denise Galloway (Fred Hutchinson Cancer Center, Seattle, USA). Recombinant retroviruses were produced from the amphotropic packaging cell line GP + envAm12 as described earlier [21]. Early passage PHKs were infected with different retroviruses at the same multiplicity of infection (MOI).

### pRb downregulation

Specific shRNAs clones for pRB silencing were selected from the MISSION® shRNA Human Gene Family Set-Tumor Suppressors (SH0531, Sigma-Aldrich, St. Louis, MO, USA) and transfected in HEK-293 T cells (ATCC® cat. no. 3216) together with MISSION® Lentiviral Packaging Mix (Sigma-Aldrich, St. Louis, MO, USA) using FuGENE® HD Transfection Reagent (Promega, Madison, WI, USA) according to the manufacturer’s instructions. Supernatants containing lentivirus were collected after 48 and 72 h after transfection. Lentiviral particles titer was determined using an HIV-1 p24 antigen enzyme-linked immunosorbent assay (ELISA) kit (ZeptoMetrix Corporation, Buffalo, NY, USA). Sub-confluent cultures of primary human keratinocytes were infected with lentiviral particles (MOI 5) expressing specific shRNA. After 2 days cells were selected with 2.5 μg/ml of puromycin (Sigma-Aldrich, St. Louis, MO, USA) until complete death of uninfected cells was observed.

### Organotypic raft cultures

Organotypic raft cultures of PHK were prepared as described elsewhere [22]. Raft cultures were allowed to differentiate for 9 days and rapamycin (Calbiochem, San Diego, CA, USA) was added to a final concentration of 100 ng/ml at day 6. Bromodeoxyuridine (BrdU) (Sigma-Aldrich, St Louis, MO, USA) was added to a final concentration of 50 μg/ml for the last 12 h. The rafts were then harvested, fixed in formalin and paraffin embedded.

### Immunohistochemistry

Raft sections were deparaffinized and dehydrated in xylene and alcohol sequential baths. The endogenous peroxidase activity was quenched with 3% H_2_O_2_ after antigen retrieval in boiling citrate buffer. Primary antibodies against BrdU (1:400; Zymed, San Francisco, CA, USA), p53 DO-7 (1:400; DakoCytomation, Glostrup, Denmark) and pRb (1:500; Novocastra, Newcastle-upon-Tyne, UK) were incubated for 18 h in 1% bovine serum albumin-phosphate buffered solution. After incubation with secondary antibody, antigens were detected with streptavidin-biotin-peroxidase complex (StreptABComplex/HRP Duet Mouse/Rabbit DakoCytomation, Dako). Chromogenic detection of peroxidase was performed with diaminobenzidine (DAB) substrate (Sigma-Aldrich, St Louis, MO, USA). The percentage of BrdU-positive/total nuclei was determined by direct counting cell nuclei. At least 3000 nuclei were counted per experiment.

### Cell proliferation assays

For Alamar blue based proliferation analysis cells silenced with lentiviral particles (MOI 5) expressing the shRNA described above and appropriate controls were seeded in octuplicate in 96 wells plates (2000 cells/well). After 72 h 10 μL of Alamar blue (Life Technologies, Carlsbad, CA) were added per well and cells were incubated at 37 °C for 4 to 7 h. After this period, Alamar Blue reduction was monitored in at 570 e 600 nm in an Epoch Microplate Spectrophotometer (Bio-Tek, Winooski, VT, USA). For growth curves, cells were seeded in low density in six-well plates and treated with rapamycin (100 ng/ml) in the next day. Cell proliferation was assessed by cell counting in the following 7 days.

### Western-blot

Total protein extracts were obtained from monolayer cultures of infected PHKs using lysis buffer (50 mM Tris-HCl, 150 mM NaCl, 1% NP-40, 0.5% Sodium deoxycholate, 0.1% SDS) supplemented with complete mini proteases inhibitors cocktail (Roche Diagnostics GmbH, Mannheim, Germany). Whole cell lysates (60 μg) were subjected to SDS-PAGE and transferred to PVDF membranes (GE Healthcare, Buckinghamshire, UK). Membranes were blocked with 5% non-fat dry milk and incubated with PLD antibody (1:300; Upstate Biotechnology, Lake Placid, NY, USA), pRB antibody (1:500; Novocastra NCL-RB-358; Leica Biosystems, Newcastle-upon-Tyne, UK), actin antibody (1:1000; Sigma-Aldrich, St. Louis, MO, USA), anti-HPV-16 E6 antibody (1:500) and anti-HPV-16 E7 antibody (1:500) from Arbor Vita Corporation (Fremont, CA, USA). The immunoreactive bands were visualized by chemiluminescence using ECL reagents (GE Healthcare, Buckinghamshire, UK).

### Measurement of phospholipase D activity

To measure PLD activity, PHK infected with different recombinant retrovirus were plated in 100 mm culture dishes and allowed to reach about 80% confluence. Cells were then pre-labeled with [^3^H] myristic acid (Perkin-Elmer, Waltham, MA, USA) (6 μCi, 30 Ci/mmol) in 4 ml of culture medium for 5 h. PLD activity was determined by measuring the formation of [^3^H] phosphatidylbutanol (PtBt), the product of PLD-mediated trans-phosphatidylation, in the presence of 0.8% 1-Butanol (Sigma-Aldrich, St. Louis, MO, USA). Lipid extraction and characterization were performed by thin layer chromatography (TLC). Briefly, lipid extracts were counted in a scintillation counter to normalize all samples. Approximately 500.000 CPM of each sample were spotted onto TLC plates (silica gel 60A, Fisher Scientific, Fair Lawn, NJ, USA). TLC were developed with the upper organic phase of a mixture containing ethyl acetate:iso-octane:glacial acetic acid:H2O (1.1:0.5:0.2:1, vol/vol) and allowed to dry before being sprayed with En3hancer Spray (Perkin Elmer, IL, USA). Phosphatidilbutanol was detected by autoradiography with Kodak BioMax MR Film (Kodak, Rochester, NY, USA).

PLD activity in control and HPV gene-expressing primary human keratinocytes after pRb downregulation was determined using the Phospholipase D Assay Kit (#700590, Cayman Chemicals, Ann Arbor, MI, USA) according to the manufacturer’s instructions.

### Statistical analysis

Statistical analyses were performed by student’s unpaired t-test using Graphpad Prism Software (Graphpad Software, La Jolla, CA). All the experiments were performed at least three times. *p*-values were considered two-tailed and significance was defined as *p* < 0.05.”

## Results

### HPV-16 E7 expression increases PLD protein expression and activity

**Fig. 1:**
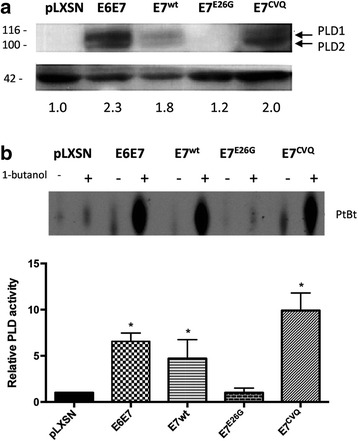
HPV-16 E7 expression increases PLD expression and activity. **a** PLD expression levels were analyzed by Western-blot in PHK transduced with empty vector, HPV-16 E6E7, wild-type E7 or E7 mutants E7^E26G^ and E7^CVQ68-70AAA^. **b** PLD activity was determined by trans-phosphatidylation reaction in the presence of 1-butanol. Generation of phosphatidylbutanol (PtBt) indicates increased PLD activity. PLD activity was normalized to that in pLXSN transduced cells, which was given an arbitrary value of 1

PLD1 and PLD2 catalyze the hydrolysis of phosphatidylcholine (PC) to phosphatidic acid (PA) and choline [23]. Due to their vital role in cell signaling and proliferation we sought to investigate if HPV-16 E7 expression could promote any alteration in PLD expression and activity. Our results show that primary human keratinocytes (PHK), expressing HPV-16 E6 and E7 or E7 alone, show an increase in both PLD1 and PLD2 isoforms when compared with keratinocytes transduced only with the empty vector (Fig. 1a). This fact was paralleled by an increase in PLD activity (Fig. 1b). E6 expression alone did not promote any alterations in PLD activation (Additional file 1: Figure S1). Interestingly, we observed that E7 ability to induce PLD expression and activation is dependent on the integrity of its LxCxE motif, as the E7^E26G^ mutant failed to induce PLD expression or increase its activity (Fig. 1a and b). An intact LxCxE motif enables E7 protein to bind pRb protein leading to the release of E2F transcriptional factor and progression into S phase [24, 25]. On the other hand, the E7 mutant (E7^CVQ68-70AAA^), that keeps the ability to destabilize pRb family members [26], was able to upregulate PLD like the WT E7 protein. Altogether these data indicates that HPV-16 E7 promotes an upregulation in PLD expression and activity and this effect is dependent on the integrity of its LxCxE motif.

### HPV-16 E7 induces PLD activation in a pRb dependent-manner

**Fig. 2:**
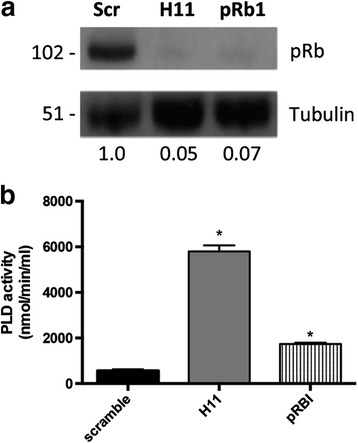
pRb downregulation leads to PLD activation in PHK. **a** Expression of pRb in PHK was silenced using lentiviral particles codifying specific shRNAs. Silencing efficiency was determined by western blot in 30 micrograms of total protein extracts. **b** The effect of pRb silencing on PLD activity was determined using Phospholipase D kit according to manufacture instructions. Error bars represent the standard deviation for three independent experiments. (*) *p* < 0.05

We investigated if pRb inactivation, induced by HPV-16 E7, could be linked to PLD increased activation. To address this question, foreskin primary human keratinocytes (PHK) were transduced with two different shRNAs targeting pRb. Retinoblastoma protein was strongly downregulated in both cell lines when compared to PHK transducing scrambled shRNA (Fig. 2a). Moreover, PLD activity was strongly upregulated in cells with pRb depleted (Fig. 2b). This result indicates that PLD activation, induced by E7, is linked to its ability to induce pRb degradation.

### HPV-16 E7 expression confers resistance to the antiproliferative effect of rapamycin

**Fig. 3:**
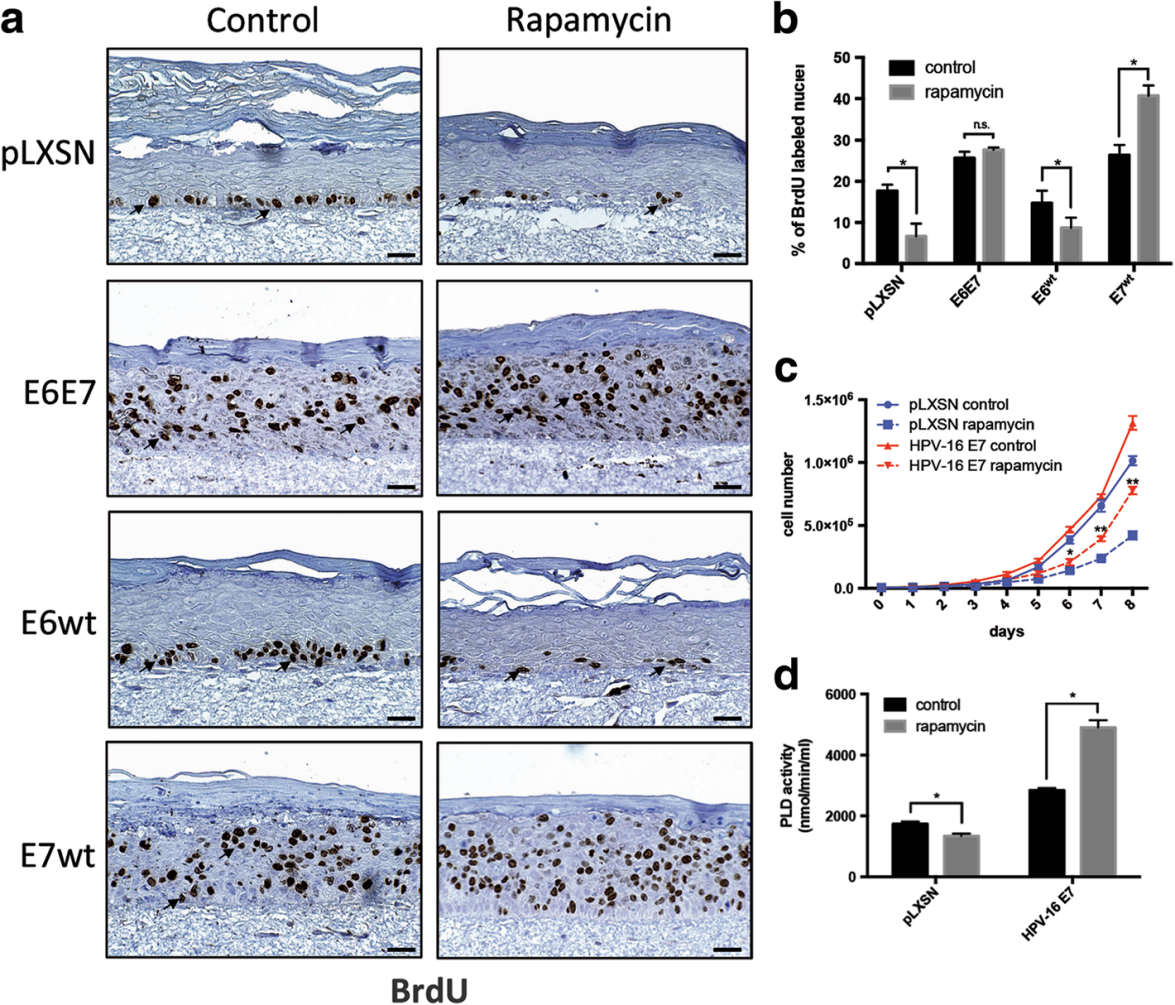
HPV-16 E7 expression confers resistance to the antiproliferative effect of rapamycin. **a** DNA synthesis in organotypic cultures of keratinocytes was detected by BrdU incorporation followed by immunohistochemistry. Arrows indicate BrdU-positive nuclei. **b** The percentage of BrdU-positive/total nuclei was determined by direct nuclei counting. The bars correspond to values obtained in at least three independent different experiments. (*) *p* < 0.05. **c** Growth curve of monolayer cultures of keratinocytes treated with 100 ng/ml rapamycin for 7 days. Results are representative of three independent experiments performed in triplicates. (*) *p* < 0.05; (**) *p* < 0.001. **d** Rapamycin treatment is associated with increased PLD activity in cultures of PHK expressing HPV16 E7. PLD activity was determined as described in Fig. 2b. Scale bars, 20 μm

Elevated PLD activity has been previously linked to rapamycin resistance [18]. Rapamycin is a highly specific inhibitor of mTOR and has been widely used to block cell proliferation [27]. Considering this, we sought to study the effect of rapamycin in the proliferation of human keratinocytes expressing HPV-16 main oncogenes in organotypic raft cultures of human keratinocytes. These cultures exhibit highly reduced p53 and pRb expression levels as an indication of the presence of functional E6 and E7 (Additional file 2: Figure S2a). HPV-16 E6 and E7 expression was also confirmed by Western-blot (Additional file 2: Figure S2b). In keratinocytes transduced with empty vector, from now on referred as control cultures, DNA synthesis was observed only in cells from the basal and parabasal layers of the epithelium (Fig. 3a). After being treated with rapamycin, control cultures showed 50% less BrdU incorporation than the untreated tissue (Fig. 3b). On the other hand, organotypic cultures of keratinocytes expressing E6E7 were significantly resistant to the antiproliferative effect of rapamycin (Fig. 3a and b). In organotypic cultures expressing only HPV-16 E6, BrdU incorporation rate decreased by 40% after rapamycin treatment (Fig. 3a and b). Surprisingly, when PHK were transduced only with HPV-16 E7 alone, rapamycin treatment promoted an increase in BrdU incorporation. Growth curve of monolayer cultures of keratinocytes treated with 100 ng/ml rapamycin for 7 days also indicate that PHK transduced with HPV-16 E7 alone proliferate much faster than PHK transduced with the empty vector (Fig. 3c). Interestingly, we also found that rapamycin treatment increased rather than decreased PLD activity in PHK expressing HPV-16 E7 (Fig. 3d). Our results show that HPV-16 E7 expression in primary human keratinocytes not only confers resistance to the antiproliferative effect of rapamycin, but also promotes proliferation in organotypic cultures expressing only this oncoprotein.

**Fig. 4:**
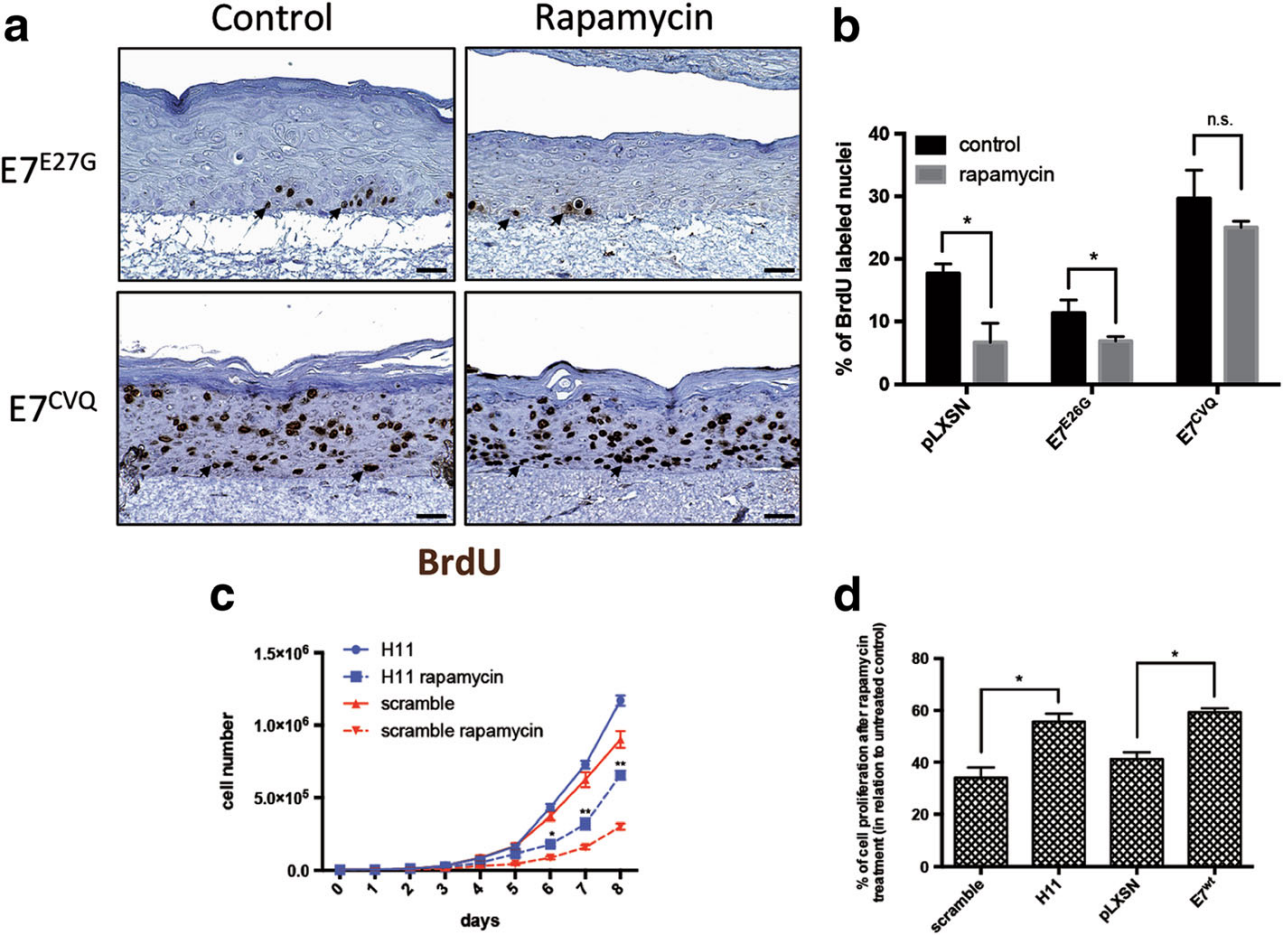
E7-mediated rapamycin resistance requires an intact LxCxE motif. **a** DNA synthesis in organotypic culture of keratinocytes expressing HPV-16 E7 mutants was detected by BrdU staining. Arrows indicate positive nuclei. **b** The percentage of BrdU-positive/total nuclei was determined by direct nuclei counting. The bars correspond to values obtained in at least three independent experiments. (*) *p* < 0.05. **c** Growth curve of monolayer cultures of keratinocytes treated with 100 ng/ml rapamycin for 7 days. Results are representative of three independent experiments performed in triplicates. (*) *p* < 0.05; (**) *p* < 0.001. **d** Alamar Blue cell proliferation assay was used to evaluate the effect of rapamycin on the proliferation of PHK silenced for pRb expression HPV16 E7 transduced PHKs. Gene silencing was performed as described in Fig. 2. Experiments were carried out in triplicate for each time point. Scale bars, 20 μm

We also investigated if the integrity of the LxCxE E7 motif could affect rapamycin resistance. Our results show that E7^E26G^ expressing cultures were as sensitive to rapamycin as control samples (Fig. 4a and b). On the other hand, DNA synthesis was not affected by rapamycin in cultures expressing the E7CVQ68-70AAA mutant (Fig. 4a and b). We also observed that PHK expressing shRNA against pRb are much more resistant to rapamycin treatment than those transduced with scrambled shRNA (Fig. 4c and d). This result indicates that rapamycin resistance observed in these cultures is associated to the ability of E7 to induce pRb degradation.

## Discussion

A growing body of evidences gathered during the past few years point toward the role of PA in mTOR activation [15]. More recently, PA was identified as a major product capable of displacing DEPTOR, a mTOR binding protein that normally functions to inhibit both mTORC1 and mTORC2 pathways [17]. The major cellular mechanism for generating PA is through the hydrolysis of phosphatidylcholine by PLD [20]. Here we show that HPV-16 E7, one of the major HPV-16 oncoproteins, is able to induce both PLD expression and activity. Interestingly, PLD has been considered a critical regulator of cell proliferation and abnormalities in its activity have been observed in many human cancers [23]. Additionally, PLD activity is elevated in cells transformed by a variety of oncogenes including *v-Src*, *v-Ras*, *v-Fps* and *v-Raf* [23]. We also demonstrate that PLD activation induced by E7 is dependent on the integrity of the LxCxE motif. This particular region of HPV-16 E7 is known to promote AKT activation in primary human keratinocytes grown in organotypic cultures [28]. The LxCxE motif of high-risk HPVs is also responsible for binding pRb leading to E2F release. For this reason it was previously linked to the ability of HPV-16 E7 to induce cellular transformation [25, 26]. Considering this, we sought to investigate if pRb inactivation could promote PLD activation. Our results show that PHK depleted of pRb present an increase in PLD expression and activity. Interestingly, E2F putative binding sites were identified in PLD1 and PLD2 promoters using the TRANSFAC database [29] and resistance to mTOR inhibitors could be found in cells with defective regulation of the retinoblastoma protein checkpoint [30]. In line with this observation and the fact that PLD overexpression also confers resistance to the mTOR inhibitor rapamycin, we investigated if cells expressing HPV-16 E7 could also display rapamycin resistance. Our results indicate that HPV-16 E7 is also capable to confer resistance to the antiproliferative effect of rapamycin. Supporting our previous observation, this resistance was also associated to the integrity of the LxCxE motif. This result is not without precedent once E7 is also capable of inducing resistance to other cytostatic agents such as TGF-β and TNF in an LxCxE motif dependent-manner [31–33].

One limitation of our study is related to the fact that exogenous PLD expression is associated to human keratinocyte differentiation [21] which precludes the possibility to test directly the effect of PLD overexpression in rapamycin resistance in normal PHK. Nonetheless, our results highlight the importance of HPV-16 E7 in bypassing negative growth regulatory signals [34].

**Fig. 5:**
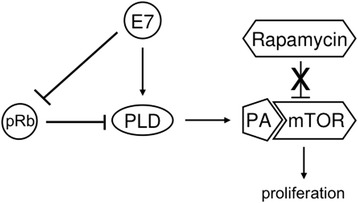
A proposed model for HPV-16 E7 induced resistance to rapamycin and PLD activation. In HPV-16 E7 expressing cells, high intracellular PA levels, generated by increased PLD activity, competes with rapamycin for mTOR binding leading to an increase in cell proliferation

Intriguingly, we found that cultures of keratinocytes expressing only HPV-16 E7 presented an increase in proliferation and an increase in PLD activity after rapamycin treatment. Although this effect was not seen in cells expressing HPV-16 E6 concomitantly, this observation could help us to understand why HPV-associated tumor xenografts generated in immuno-compromised mice grow slower when rapamycin is administered daily to the animals, but fail to lead to any long-term cure [35]. Even with the treatment prolonging survival and delaying cell proliferation, tumors cells could still grow ultimately affecting survival [35]. For this reason, several studies are suggesting the use of rapamycin in combination with other therapeutic drugs [36]. In fact, rapamycin and rapamycin derivatives are being proposed as a concurrent agent to standard-of-care cisplatin/radiation therapy to attenuate tumor lactate production and induce regression of (HPV)-related head and neck squamous cell carcinomas (HNSCC) [36]. A proposed model describing our findings is presented in Fig. 5.

## Conclusions

Our work present evidences that HPV-16 E7 up-regulates PLD activity. It also shows that the increase in PLD activation is related to the ability of E7 to induce pRb degradation. Moreover, we show that cells depleted of pRb expression exhibit higher PLD activity. Supporting our findings we present data indicating that both HPV-16 E7 expression and pRb depletion lead to resistance to the antiproliferative effect of rapamycin. Considering the fact that rapamycin and rapamycin analogs are being combined with other chemotherapeutic drugs, it is possible that rapamycin may be associated with PLD inhibitors to circumvent rapamycin resistance exhibited by many types of human cancers, including those related to HPV. Further studies are warranted.

## Additional files

Additional file 1: **Figure S1.** HPV-16 E6 did not affect PLD activity. (PDF 127 kb)

Additional file 2: **Figure S2.** HPV-16 E6 and E7 expression affect p53 and pRb expression. (PDF 116 kb)

## Abbreviations

BPE: Bovine pituitary extract
Brdu: Bromodeoxyuridine
DAB: Diaminobenzidine
HNSCC: Head and neck squamous cell carcinomas
HPV: Human Papillomavirus
MOI: Multiplicity of infection
mTOR: Mammalian target of rapamycin
mTORC1: MTOR complex 1
PA: Phosphatidic acid
PC: Phosphatidylcholine
PHK: Primary human keratinocytes
PI3K: Phosphatidylinositol (PI)-3-kinase
PLD: Phospholipase D
pRb: Retinoblastoma protein
PtBt: Phosphatidylbutanol
