# Supplementary material for: HPV-16 E7 expression up-regulates phospholipase D activity and promotes rapamycin resistance in a pRB-dependent manner
Source: BMC Cancer. 2018 Apr 27;18:485. doi: 10.1186/s12885-018-4392-8 (PMC5923196; doi:10.1186/s12885-018-4392-8)
Supplement: Supplementary file 1 — Figure S1. HPV-16 E6 did not affect PLD activity. (PDF 127 kb) [file 12885_2018_4392_MOESM1_ESM.pdf]

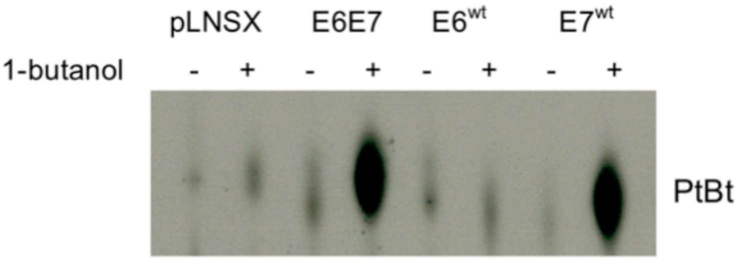

Figure S1. HPV-16 E6 did not affect PLD activity. Autoradiogram showing the result of Thin-Layer Chromatography where radioactive phospholipids were separated to monitor the generation of phosphatidil butanol, an indicative of strong PLD activity.
