# Supplementary material for: HPV-16 E7 expression up-regulates phospholipase D activity and promotes rapamycin resistance in a pRB-dependent manner
Source: BMC Cancer. 2018 Apr 27;18:485. doi: 10.1186/s12885-018-4392-8 (PMC5923196; doi:10.1186/s12885-018-4392-8)
Supplement: Supplementary file 2 — Figure S2. HPV-16 E6 and E7 expression affect p53 and pRb expression. (PDF 116 kb) [file 12885_2018_4392_MOESM2_ESM.pdf]

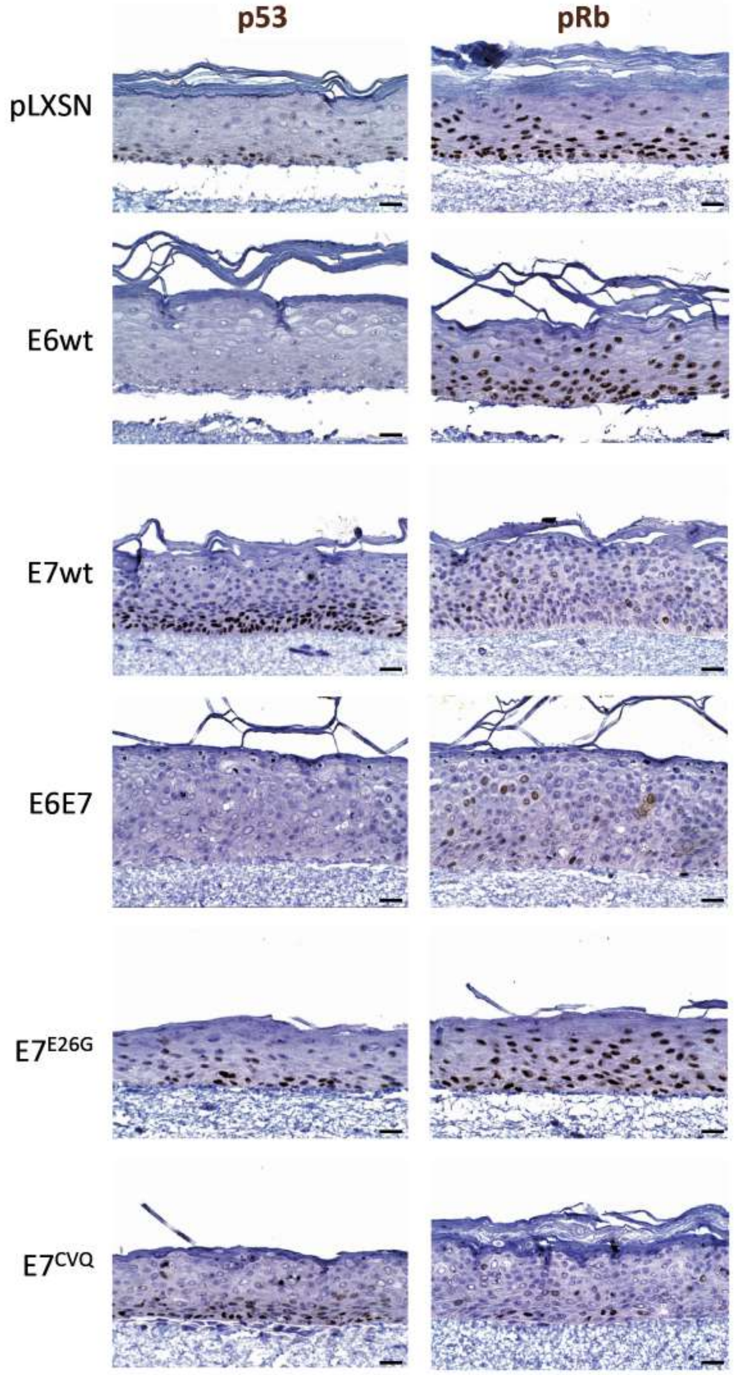

Fig. S2. E6 and E7 expression affect p53 and pRb expression in primary human keratinocytes. Immunohistochemistry indicating p53 and pRb staining in organotypic cultures of keratinocytes expressing HPV-16 WT genes and E7 mutants. Scale bars, 20µm.
